# Parathyroid hormone secretion by multiple distinct cell populations, a time dynamic mathematical model

**DOI:** 10.1002/phy2.231

**Published:** 2014-02-10

**Authors:** William A. Pruett, Robert L. Hester

**Affiliations:** 1 Department of Physiology, Center for Computational Medicine, University of Mississippi Medical Center, Jackson, 39216, Mississippi

**Keywords:** Calcium, hysteresis, parathyroid hormone, simulation

## Abstract

The acute response of parathyroid hormone to perturbations in serum ionized calcium ([Ca^2+^]) is physiologically complex, and poorly understood. The literature provides numerous observations of quantitative and qualitative descriptions of parathyroid hormone (PTH) dynamics. We present a physiologically based mathematical model of PTH secretion constructed from mechanisms suggested in the literature, and validated against complex [Ca^2+^] clamping protocols from human data. The model is based on two assumptions. The first is that secretion is a fraction of cellular reserves, with the fraction being determined by the kinetics of [Ca^2+^] with its receptor. The second is that there are multiple distinct populations of parathyroid cells, with different secretory parameters. The steady state and transient PTH secretion responses of the model are in agreement with human experimental PTH responses to different hypocalcemia and hypercalcemia stimuli. This mathematical model suggests that a population of secreting cells is responsible for the PTH secretory dynamics observed experimentally.

## Abstract (precis)

We present a physiologically based mathematical model of parathyroid hormone (PTH) secretion constructed from mechanisms suggested in the literature, and validated against complex [Ca2+] clamping protocols from human data. The steady state and transient PTH secretion responses of the model are in agreement with human experimental PTH responses to different hypo and hypercalcemia stimuli. This mathematical model suggests that a population of secreting cells is responsible for the PTH secretory dynamics observed experimentally.

## Introduction

Calcium homeostasis is accomplished by a complex network of interacting systems – bone, gut, kidney, and endocrine – operating on a range of timescales from minutes to months. Parathyroid hormone (PTH) is the prime hormonal determinant of calcium metabolism in humans. Acutely, PTH acts by decreasing renal excretion of calcium and increasing excretion of phosphate (Agus et al. 1973), as well as by enhancing mineral extraction from bone (Lorenzo et al. 1983). Chronically, PTH enhances 1*α*,25(OH)_2_ Vitamin D (calcitriol) production (Murayama et al. 1999) and controls the ratios of cells that produce (osteoblasts) and resorb (osteoclasts) bone (Peterson and Riggs 2010). This regulates calcium reserves and maintains a range of responses reflective of chronic conditions.

Brown (1983) published a model of the [Ca^2+^]‐PTH relationship based on data from dispersed parathyroid cells, deeming four parameters necessary and sufficient to describe the sigmoidal steady state relationship between [Ca^2+^] and PTH. The model is empirical and does not suggest mechanisms by which the particular parameters, secretion minimum and maximum, sensitivity, and set point, can be explained or predicted by known physiology. Furthermore, the model only predicts the steady state behavior of PTH with respect to calcium, not transients.

Many experiments have demonstrated a different relationship between [Ca^2+^] and [PTH] depending on whether the [Ca^2+^] is increasing or decreasing. Collectively, we refer to all deviations in the [Ca^2+^]‐[PTH] relationship as hysteresis. Many explanations have been offered for the behavior; the most widely proposed hypothesis asserts that the calcium sensing receptor (CaSR) detects the direction and rate of changes in serum calcium level and responds to those (Grant et al. 1990; Kwan et al.^1993^; Schwarz 1993; De Cristofaro et al. 2001). There has not been presented any proposed mechanism for such detection. Our purpose in this study was to construct a dynamic model of physiologically relevant mechanisms that would explain the hysteretic response.

Two dynamic models of PTH have been proposed. The first, proposed by Momsen and Schwarz was specifically designed to show that hysteresis could be partially attributed to the storage ability of the parathyroid gland (Momsen and Schwarz 1997). The second, proposed by Shrestha et al.'s (2010) group, asserted that a bisigmoidal function, that is, a logistic function whose sensitivity changes at a point near the set point of the mechanism, would better fit experimental data than the sigmoidal function proposed by Brown. Both models failed to adequately explain hysteresis in terms of storage, and Shrestha was unable to interpret the physiological significance of the secretion function's asymmetry.

Because of the lack of direct evidence for a rate‐dependent secretion mechanism, we sought other mechanisms that might produce hysteresis and be physiologically relevant. Two conclusions drawn from experiments and models suggested an explanation. First was Shrestha's observation that a standard sigmoidal function could not be parameterized so as to fit responses to both hypocalcemic and hypercalcemic stimuli (Shrestha et al. 2010). Their group's solution was to introduce an asymmetry in the sigmoidal secretion function, but without a physiological explanation of the asymmetry. Sun observed that individual parathyroid cells responded heterogeneously to a given calcemic perturbation (Sun et al. 1993). This led us to infer that the asymmetric sigmoid used by Shrestha to simultaneously fit hypercalcemic and hypocalcemic challenges could be the result of multiple populations of parathyroid cells, each responding differently to a given perturbation. This supposed heterogeneity is consistent with the working hypothesis underlying current understanding of pulsatile PTH secretion (Schmitt et al. 1996).

Heterogeneity became the basis of the model we present here. Each cell population is defined by a collection of parameters defining production, intracellular degradation, and secretion. These characteristics are randomly assigned and held static for the duration of the model. Experiments have shown that PTH is released in a pulsatile fashion (Silver et al. 1986), likely as a result of complex intercellular network dynamics. For clarity, we opted to describe secretion as a smooth function of [Ca^2+^], in effect showing the expected value of a probabilistic secretion operator. In addition, our model utilizes the storage hypothesis previously modeled by Momsen and Schwarz (1997). The endpoint for this study is a demonstration of a model composed of loosely defined rules (distributed parameters) that define secretion kinetics and account for hysteresis through a natural production/storage mechanism.

## Material and Methods

To create the model, we assumed that the secretion mechanisms were the same between all cell populations, but parameters (model coefficients) would be different. We describe the secretion mechanism first, followed by stipulating how the cell populations differ.

PTH secretion is inhibited by [Ca^2+^] interacting with CaSR on the parathyroid cell membrane. We calculate secretion as a percentage of stored PTH. Ionized calcium inhibits secretion by activating the CaSR. We model this with standard dose–response curve, that is, a decreasing sigmoidal curve:
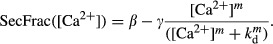
*β* represents the maximal fraction of the intracellular PTH pool capable of being secreted in a given minute, *γ* the maximal inhibition, and *m* represents an “augmentation factor”. It should be noted that this is not receptor kinetics, and *m* is not a Hill number. In particular, CaSR is believed to have four binding sites and hence a Hill number <4, while the dose–response relationship is highly variable between normal and pathological states (Brown 1983). Here, *m* represents the end result of the intracellular signaling cascade. We currently view the mechanism of inhibition as a black box problem. Secretion is never entirely repressed (Brown 1983), demonstrating the physiological importance of the difference *β*–*γ*.

We assume that PTH is produced in chief cells at a fixed rate (Morrissey and Cohn 1979), although the literature is inconsistent (Liu et al. 1994; Silver et al. 1999; Ritter et al. 2008). The rate is likely related to circulating factors such as calcitriol, phosphate, and FGF‐23 (Russell et al. 1986; Silver et al. 1986, 1999; Naveh‐Many et al. 1999). As those factors were not considered in this model, we chose to have a constant intracellular production (*k*_v_) in each cell population, though the value was allowed to differ between populations during model calibration. A portion of intracellular PTH is proteolyzed before secretion (Morrissey and Cohn 1979). We use *k*_deg_ to express the percentage of stored PTH degraded each minute; we have fixed this quantity despite some evidence that the mechanism is dependent on intracellular, and therefore extracellular, calcium. Together with functional secretion fraction, these parameters determine VPTH, the intracellular (vesicular) storage of PTH. This model does not explicitly include parathyroid gland size or storage capacity, but these factors are implicit in the production and degradation factors that determine steady state VPTH. With this, intracellular PTH is calculated as the solution to the differential equation
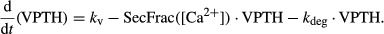


Serum PTH is calculated as the difference between secretion and clearance (*k*_cl_). PTH is cleared in the kidney and in the liver. In humans, the mean arteriovenous difference is 44% across the liver and 34% across the kidney (Hruska et al. 1981). There may be some clearance across the bone or muscle (Oldham et al. 1978), but we only considered renal and hepatic clearances in our model. Renal flow rate is assumed 0.625 L/min, and liver plasma flow rate is fixed at 1.02 L/min. We define *k*_cl_ = (RBF · *k*_cl,renal_) + (LBF · *k*_cl,hep_).

The interstitial fluid pool buffers PTH secretion. We assume that in all patients, the interstitial fluid volume is 11 L, and allow half the concentration difference to exchange between compartments per minute. The model was not sensitive to this proportion. We define the interstitial compartment PTH mass as
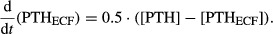


Together, these factors yield a differential equation describing changes in the serum PTH pool:
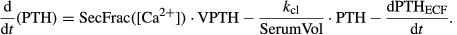


Equations 1, 2, and 4 can be solved simultaneously for steady state solution, yielding
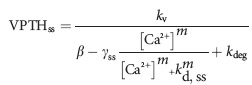

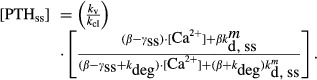


This completes the description of the model equations.

**Figure 1.**
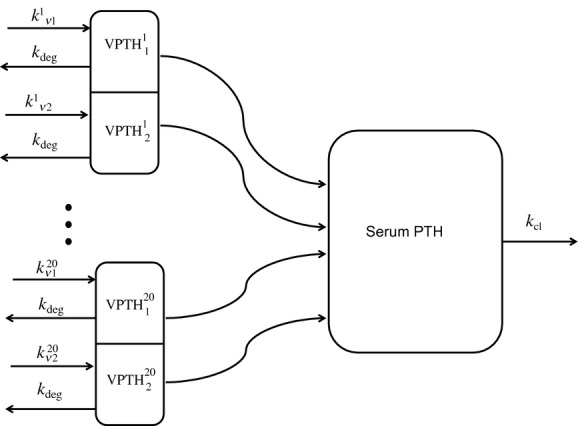
Model schematic. Each of the 20 subpopulations (denoted by superscripts 1, … 20) of parathyroid cells is subdivided into a sensitive and insensitive population (denoted by subscripts 1 and 2). Each subpopulation type has its own intracellular pool (VPTH) and secretion fraction (curved arrow), but there is only one serum pool.

Rather than using a single set of parameters to define the model, we sampled parameters from calibrated distributions calculated from a single complex calcium profile. This model philosophy assumes that all cell populations would be active, with their particular activity determined by extracellular [Ca^2+^] via the population value for *k*_d_. In particular, we calculated parameter distributions for the coefficients in the two cell populations and sampled 20 cells of each type to comprise the parathyroid gland. Each cell type would have its own storage and secretion function, with all secreted PTH being partitioned between the serum volume (3 L) and interstitial fluid volume (11 L) (Fig. 1). Physiologically, this model can be interpreted as a group of cells operating under a loose collection of rules, in contrast to a single set of parameters defining model response.

The literature suggested parameters that might vary between the cell populations. Sensitivity was assumed to be correlated with CaSR expression; mechanistically this corresponds to the probability of a cell secreting at a given calcium concentration. We refer to the cells with higher *k*_d_ as sensitive, and the rest as insensitive. This is due to the fact that, at normal [Ca^2+^], the cells with higher *k*_d_ are secreting. Because PTH mRNA expression changes with [Ca^2+^], we assumed that *k*_v_ should differ between populations (Liu et al. 1994; Silver et al. 1999). Standard receptor kinetics implies that lower expression of CaSR on the cell membrane decreases the signal the receptor is able to pass even while it lowers the amount of [Ca^2+^] necessary to elicit a response. This would imply that lower CaSR leads to decreased inhibition of secretion (*γ*), and increases the sensitivity (*m*) of the response (Cetani et al. 2000; Yano et al. 2003). This implies that a population of insensitive cells will have low values of *γ* and *m*, while a sensitive population will have the opposite characteristics. We allowed maximal secretion *β* to vary between cells, but used the same distribution for sensitive and insensitive cells. There is conflict in the literature regarding [Ca^2+^] influence on *k*_v_ (Morrissey and Cohn 1979; Liu et al. 1994; Naveh‐Many et al. 1999; Silver et al. 1999); we chose to let *k*_v_ be independently generated between sensitive and insensitive populations. While some evidence has been presented that indicates that the degradation constant *k*_deg_ is greater in sensitive cells than in insensitive ones (Habener et al. 1975), this can be counterbalanced by changes in *k*_v_. For this reason, we chose to let *k*_v_ manifest the variability in the storage pool (VPTH), and fix *k*_deg_ = 0.02, corresponding to a decay rate of 70% in 60 min as reported by Morrissey and Cohn (1979) group.

Because the model philosophy involves the construction of multiple populations of cells, we required distributions describing each parameter for the insensitive and sensitive cells types. The model was calibrated by fitting baseline, hypocalcemic and hypercalcemic responses, as well as a single hysteretic response as reported in a single clamping protocol for a group of four individuals by Schwarz et al. (1998). It was composed of hypocalcemia (−0.20 mmol/L from baseline) induced for 75 min, followed by a brief round of hypercalcemia (+0.20 mmol/L from baseline), followed by reinduction of hypocalcemia (−0.20 mmol/L from baseline) (Protocol 1 in the reference). We collected from these experiments the data points used to generate the experimentally observed distribution of [PTH] using the SmoothKernelDistribution function of Mathematica at normocalcemia, hypocalcemia, and hypercalcemia, as well as the peak response during induction of hypocalcemia. To calibrate the population parameters to generate output distributions in accord with observations, we assumed initial uniform distributions for each parameter, sampled 2500 models and jackknifed the model output to retain only those models whose outputs were within 1.5 SD of the experimentally recorded output. We assumed that each output was independent. This resulted in a reduced number of models/parameter combinations that were then used to estimate new distributions for each parameter via the SmoothKernelDistribution function. This process continued until the output data established a sufficiently high goodness of fit against the experimental data. The calibrated parameter distributions were used for all model generation. Because each parameter was one‐dimensional, sampling was accomplished by inverting the cumulative distribution constructed with smooth kernel methods.

Model output and experimental data were compared in the following way. In the experimental protocol used for calibration, one time point corresponded to the baseline [PTH], five time points corresponded to [PTH] in response to hypocalcemia, two to the initial hysteretic response, and three to hypercalcemia. The PTH values at these approximations of steady state were pooled, and *z*‐scores were computed relative to the experimental mean. The outputs were considered jointly sufficiently normal provided the average z‐score product was less that 0.1, and the maximum *z*‐score was smaller than 0.75.

**Figure 2.**
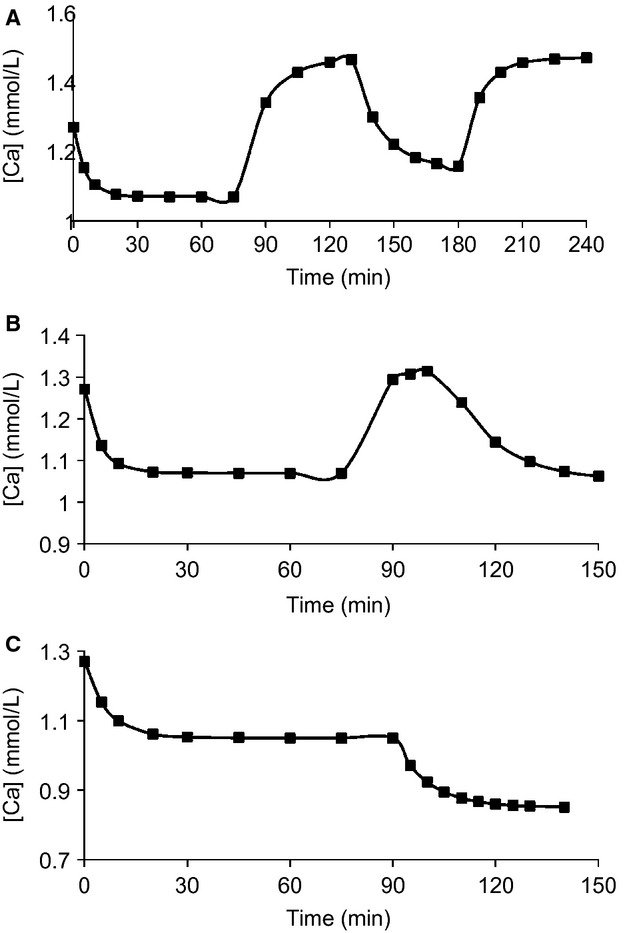
Ideal calcium profiles in the training and validation protocols defined in Schwarz et al. (1998). (A) Curve used for training the responses; (B and C) curves used in validation. All calcium measurements are given in mmol/L.

The model is validated against the other two experiments reported by Schwarz et al. (1998). The first was a complex clamp protocol composed of an initial hypocalcemic clamp (−0.20 mmol/L from baseline) for 100 min, followed by a brief return to normocalcemia, followed by another episode of hypocalcemia (−0.20 mmol/L from baseline). The second protocol involved an initial hypocalcemia (−0.20 mmol/L from baseline) for 60 min followed by an extreme hypocalcemic clamp (−0.40 mmol/L from baseline). Individual calcium curves differed from the ideal curves defined in the experimental protocol. We used the ideal curves to generate the model response; these are shown in Figure 2.

The model can be fully downloaded at http://hummod.github.com/pth-model-transient. It requires Mathematica 8 (www.wolfram.com).

## Results

The calibration algorithm yielded parameter distributions with means and deviations shown in Table 1. The calibrated distributions were not sensitive to the initial distributions chosen, and multiple calibrations yielded statistically similar distributions. We utilized uniform distributions for all initial calculations to avoid bias. Rather than making any assumptions about what the parameter distributions might be, we used smooth kernel methods to construct distributions from retained models.

**Table 1.** Model parameters with descriptions and calibrated search ranges.

Parameter	Initial distribution	Postcalibration mean and standard deviation	Description
*k*_v1_	U[1, 10]	(3.6, 1.13)	Intracellular PTH synthesis (mmol)
*k*_v2_	U[1, 10]	(2.1, 1.41)	
*β*	U[0.1, 0.9]	(0.50, 0.26)	Maximum percent of secretion from intracellular pool (unitless)
*γ*_*c*_	U[0.00005, 0.0025]	(0.0032, 0.00117)	Minimal fractional secretion as a percentage of total intracellular pool. *γ = β – γ*_*c*_ (unitless)
*m*_1_	U[100, 400]	(270, 102)	Sensitivity of the black box inhibition function (unitless)
*m*_2_	U[100, 400]	(244, 92)	
*k*_d1_	U[1.025, 1.175]	(1.13, 0.056)	Set point of the black box inhibition function (mmol/L)
*k*_d2_	U[1.20, 1.35]	(1.27, 0.051)	

PTH, parathyroid hormone.

**Figure 3.**
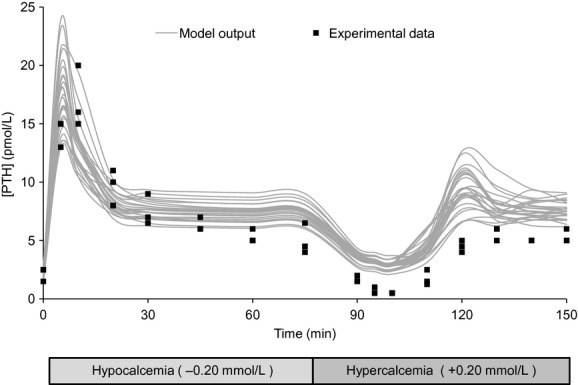
Model validation results: validation of the population model against the hypocalcemia‐normocalcemia‐hypocalcemia protocol as reported by Schwarz et al. (1998). The gray curves denote 25 model outputs, while the black boxes show the individual data redrawn from Schwarz. The calcium curve corresponding to this protocol is Figure 2B.

The model response to the hypocalcemia‐normocalcemia‐hypocalcemia clamping protocol is shown in Figure 3. The model results were highly coherent with experiment, matching the initial hysteresis, hypocalcemic steady state, and second peak very well. The model consistently overpredicted the response to normocalcemia. In repeated runs, 37 ± 4.5% of the experimental data points lay within the pointwise 95% confidence interval predicted by the model. The dynamic response differed between model and experiment at only three of 16 time points; at each, the model predicted near a fall or rise of less than 0.5 pmol/L.

**Figure 4.**
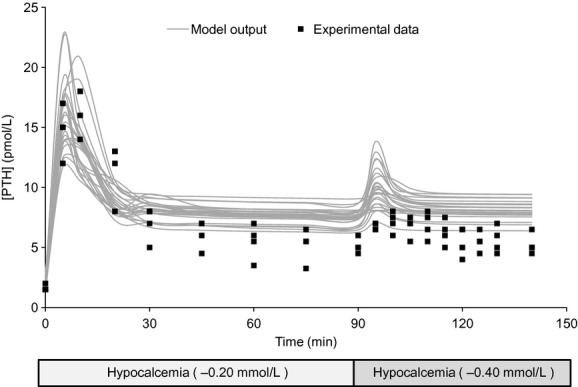
Model validation results: validation of the population model against the hypocalcemia‐extreme hypocalcemia protocol as reported by Schwarz et al. (1998). The gray curves denote 25 model outputs, while the black boxes show the individual data redrawn from Schwarz. The calcium curve corresponding to this protocol is Figure 2C.

The model response to the hypocalcemia‐extreme hypocalcemia clamping protocol is shown in Figure 4. Pointwise confidence intervals were constructed based on 25 model outputs, and compared to experimental results. In repeated runs, 44 ± 9.3% of experimental data points lay in the model's 95% confidence interval. In contrast to the first validation, the data points that lay outside the confidence intervals were distributed more evenly through the protocol. The overall dynamic trends of the protocol were similar to that recorded in humans, being correct at 14 of 18 time points. The differences were concentrated in the late second peak, where experimental subjects experienced some oscillation and the model was more stable. Also of note, when only two cell populations were used (one sensitive and one insensitive), the second peak did not develop. It appears to be an artifact of [Ca^2+^] descending below the sampled values of *k*_d_ in more insensitive cells, rather than an implicit part of the system dynamics.

We compared our model with existing steady state and dynamic models. Steady state was analyzed in two ways: the slope of the [Ca^2+^]‐[PTH] relationship and the parameterized sensitivity of the relationship. After solving our model for steady state in each individual, we computed the linear slope of the relationship by calculating the slope between 10% and 90% maximal secretion. We obtained 329 ± 35%/(mmol/L) as compared to Malberti's observation of 475 ± 86%/(mmol/L) (Malberti et al. 1999) and Messa's value of 395 ± 150%/(mmol/L) (Messa et al. 1994). The steady state curve was fit to a four parameter model as in Brown (1983); we observed a sensitivity of 32, whereas Brown observed a much smaller value of 7.5 in a normal population.

**Figure 5.**
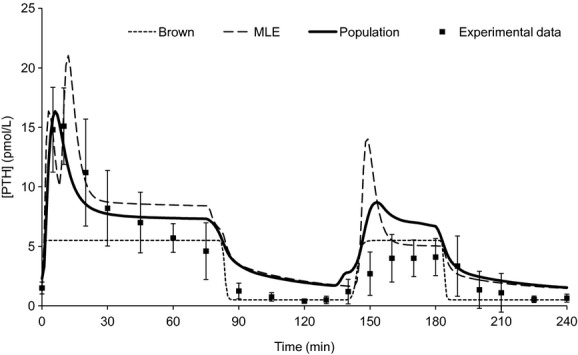
Comparison of Brown's model (dotted line), a model consisting of a single pair of insensitive and sensitive cells (dashed line), and a model composed of 20 such pairs to the calibration protocol (solid line). The experimental output that generates this output is shown in black boxes with standard deviation bars shown. The calibration used a subset of this data, namely the responses at 0 min (baseline), 5 and 10 min (initial hysteretic response), 30–60 and 150–170 min (hypocalcemic steady state), and 90–120 min (hypercalcemic steady state). The calcium curve generating this output is given by Figure 2A.

Finally, to demonstrate the superior dynamics displayed by the model, we fit Brown's (1983) four parameter description of serum [PTH] () to the baseline, hypocalcemic, and hypercalcemic responses in the calibration protocol. Similarly, we fit the “single population” model (one sensitive and one insensitive cell population) to a single set of parameters, using baseline, hypocalcemic and hypercalcemic response, and initial hysteretic response as in the calibration step. The outcome of these models is compared to the population model in Figure 5. Quantitatively, the multiple population model outperforms the other models using squared fit residuals as the metric (125 vs. 148 and 320 in Brown's model and the single population model, respectively). Qualitatively, Brown's model performs well primarily due to the fact that 11 of the 22 examples were drawn after [Ca^2+^] had equilibrated to the steady state; this model showed no hysteresis. The single population model showed a fair approximation of the dynamics, but due to the width of the parameter intervals, the output curve was inconsistent between runs.

## Discussion

In this paper, we present a model of the PTH response to an array of complex serum calcium clamps. There are two main features of this model. First, the model provides a natural physiological interpretation of each parameter, as opposed to the empirical parameters developed previously. Secondly, the model suggests that the total parathyroid hormone response is the result of multiple populations of cells obeying a loosely defined (parameterized) set of rules in tandem with one another. This assumption generates transient PTH curves in close agreement with experimental data, in particular explaining the observed hysteresis without requiring rate‐dependent secretion (Fitzpatrick and Leong 1990; Cetani et al. 2000).

In order to develop an integrative, adaptive model of calcium homeostasis, it is necessary to develop a model whose parameters are themselves functions of pathological state and serum factors. Each of the parameters in such a model must then represent physiologically interpretable quantities, in particular the known factors that control production, degradation, and secretion of PTH. Previous models have concentrated on empirically observable aspects of PTH secretion: the maximum and minimum levels of secretion, the [Ca^2+^] correlated with half‐maximal PTH response, and the sensitivity of the response. While these factors are worthy of study, they do not add to the knowledge of the mechanisms that determine circulating PTH. This study was undertaken to provide the basis for an adaptive model.

The population assumption was the most critical one in the model. It followed from several observations from the literature, as well as the fact that no current model of the PTH‐[Ca^2+^] relationship can be parameterized in a way that matches experimental observations of the PTH transient during the onset of complex calcium clamps. By proposing a model of two different sensitivities in the [Ca^2+^]‐PTH relationship, Shrestha's paper implicitly suggested that either there were at least two distinct populations of parathyroid cells or that sensitivity was itself [Ca^2+^]‐dependent. Ideally, a model of PTH transients would involve cells switching between active and inactive states; this phenomenon was observed by Sun et al. (1993), and could explain the phenomenon of pulsatile secretion noted by Schmitt et al. (1996). Switching in a complex dynamical system requires the use of latent variables that we felt could not be validated. Therefore, we adopted the use of two distinct types of cells, and sampled 20 examples from each type to make each individual. We chose the 50–50 partition between the sensitive and insensitive types based on data obtained from reverse hemolytic plaque techniques (Fitzpatrick and Leong 1990; Ritchie et al. 1992; Sun et al. 1993, 1994). The model performance was not sensitive to that particular proportion. The decision to use 20 cell populations per individual was arbitrary; fewer populations showed an irregular jagged response not correlated with observed switching behavior and more populations showed a smoother response. The populations were assumed to differ only in their production of PTH, their sensitivity to PTH, and the set point for the secretory mechanism. After the parameter distributions were calibrated, sensitivity was seen to be statistically similar in each population, with production and the set point significantly different between populations.

The population hypothesis can be interpreted in several ways in light of experimental observations. Our primary interpretation is that the parathyroid glands are composed of multiple colonies of cells similar secretory characteristics, perhaps due to gap‐junction connections between cells. An alternate interpretation is that two distinct types of vesicles are present in each cell, and their secretory characteristics differ with respect to the intracellular signal cascade initiated by the CaSR. The parameterizations obtained would support the conclusions of Setoguti, who observed that in the rat, parathyroid storage granules exocytose their contents when extracellular [Ca^2+^] falls below 1 mmol/L (Setoguti et al. 1995).

We have made several simplifying assumptions about the general relationship between PTH and [Ca^2+^]. The first is that serum calcium is an independent variable with respect to PTH wholly determined by the ideal laid out in the experimental protocol. Decoupling of the [Ca^2+^]‐PTH feedback loop prevents the demonstration of lag hysteresis (Schwarz et al. 1998) but avoids the problem of establishing quantitatively the effects of PTH on calcium metabolism. In addition, it leaves our model predictions sensitive to our representation of [Ca^2+^], for example, in the polynomial order chosen for interpolation. Furthermore, it introduces error into the model output by not imitating exactly the individual calcium curves obtained experimentally. Similarly, we do not model any of the myriad non‐[Ca^2+^] factors known or believed to modulate PTH secretion, among them calcitriol status (Silver et al. 1986, 1999) and serum phosphate (Almaden et al. 1996). In vivo, these factors are intertwined; changes in one rarely happen without commensurate changes in the others. The chief reason for not including them is that the published reports generally contain information only about baseline values of calcitriol and phosphate. Finally, we assume that PTH synthesis reflects a finished product; we are not considering transcriptional or any posttranscriptional modifications, or the time delays that have been previously observed (Silver et al. 1986; Hinton and MacGregor 1991; Almaden et al. 1996; Ben‐Dov et al. 2007; Galitzer et al. 2008). In particular, we chose to model intracellular degradation of PTH as a constant, in opposition to some observations concerning in vitro [Ca^2+^]‐sensitivity (Habener et al. 1975; Morrissey and Cohn 1979) and in vivo (Schwarz et al. 1992; Bas et al. 2002).

We tested the calibrated model against two complex calcium clamping protocols with two endpoints: first, the performance of the individual experimental results compared to 95% confidence intervals established by the model, and second, the discrete dynamic correctness of the model response compared to the experimental data. The purpose of the discrete test was to evaluate the model hysteretic response in a qualitative manner. The simplifying assumptions detailed above introduced uncertainty into the models qualitative response that is difficult to evaluate. By assuring that the direction of the response was correct, we provide a secondary validation of the model.

The model provided a quantitative response consistent with experimental observations. Although the actual percentage of experimental points that fell within the model predicted 95% confidence intervals was lower than we would have hoped, there are reasonable explanations. First, we limited the calibration algorithm to assume independence among the output distributions. This was primarily a technical choice due to the small number of data points. Secondly, we made no effort to shape the calibration distributions other than to align the means of the outputs with the means observed experimentally. This was also a modeling choice, and was made primarily for the same reason as above. Finally, we assumed that each individual manifested the same calcium profile, while the individuals in the experiment had noisy varying responses to the clamp. We felt that it was more reasonable to show the response to a controlled fixed curve than to try to imitate the individual responses seen in each patient.

Qualitatively, the model also performed very well. In all cases, discrepancies between model output and the experimental results occur when the magnitude of the change was very small. The majority of points showing incorrect dynamics occurred during the recovery from a hysteretic peak, which suggested that cell populations switching into and out of secretory phase could play a role in the dynamic discrepancies. As above, the choice to use ideal calcium curves instead of explicitly imitating the actual calcium responses could have played a role in the differences in our quantitative measures. In the period of decline from a peak patients showed a slower decline than the model predicted; we believe that this is related to two decisions made during model production: the decision not to model phosphate homeostasis and to leave *k*_v_ constant. By removing the secondary PTH secretagogue and leaving production constant, our predictions should be expected to diverge from experimental observations.

We have constructed a model of human PTH secretion that produces results that qualitatively and quantitatively agree with experimental observations. The model is novel in that the response to a given stimulus is based on the average response of a large number of independently parameterized subpopulations. This factor enables it to produce hysteresis in calcium clamp protocols that demonstrate fidelity to observations seen in normal humans.

## Conflict of Interest

None declared.
